# Clinical Features and Seroepidemiology of Anti-HDV Antibody in patients With Chronic Hepatitis B Virus Infection in Iran: A Meta-Analysis

**DOI:** 10.5812/kowsar.1735143X.805

**Published:** 2011-12-20

**Authors:** Neda Amini, Seyed Moayed Alavian, Ali Kabir, Seyed Yaser Saiedi Hosseini, Seyed Hossein Aalaei Andabili

**Affiliations:** 1 Tehran University of Medical Sciences, Students’ Scientific Research Center, Tehran, IR Iran; 2 Baqiyatallah Research Center for Gastroenterology and Liver Diseases, Baqiyatallah University of Medical Sciences, Tehran, IR Iran; 3 Tehran University of Medical Sciences, Center for educational Research in Medical Sciences, Tehran, IR Iran; 4 Department of epidemiology, Shahid Beheshti University of Medical Sciences, Tehran, IR Iran

**Keywords:** Hepatitis D, Review, Meta-Analysis, Iran, Revalence, Epidemiology

## Abstract

**Context:**

Hepatitis delta virus (HDV) leads to the most severe form of chronic viral hepatitis.

**Objectives:**

To determine the prevalence of HDV and create pooled estimations of possible risk factors, a systematic review was conducted to collect all epidemiological studies on HDV among chronic hepatitis B patients in Iran.

**Data Sources:**

In this systematic review, databases such as PubMed, Embase, ISI, Google scholar, and Iranian databases (MagIran, Iranmedex, and SID) were searched.

**Study Selection:**

Studies that clearly stated information about the number of HBsAg positive patients infected with HDV were selected.

**Data Extraction:**

The name of the city, the author's name, year of study, HDV detection method, sample size, HBsAg positive frequency, mean age, total prevalence of HDV, and risk factors were extracted.

**Results:**

The pooled HDV prevalence was 7.8% (95% CI: 5.89 - 9.71). In the survey-data analysis, HDV prevalence was 6.61%. HDV prevalence was 30.47% (95% CI: 9.76 to 51.19), 14.4% (95% CI: 7.72 to 21.07), and 4.94% (95% CI: 3.73 to 6.15) in cirrhotic, chronic-hepatitis, and inactive-carrier patients, respectively. Pooled ORs were calculated for several factors common to Iranian HBsAg-positive patients, including history of blood transfusion [OR: 1.1 (95% CI: 0.40 to 2.98)], intravenous drug abuse [OR: 1.6 (95% CI: 0.78 to 3.21)], previous hemodialysis [OR: 1.72 (95% CI: 0.79 to 3.76)], and HBeAg-positive status [OR: 1.26 (95% CI: 0.66 to 2.4)].

**Conclusions:**

The prevalence of HDV is less common in Iran than in endemic regions such as Italy and Turkey; however, it is a severe form of hepatitis in HBsAg-positive patients. The most probable route of HDV transmission is hematologic, which suggests the importance of blood screening for HDV, especially in groups with numerous blood transfusions.

## 1. Context

Delta hepatitis infection leads to the most threatening form of chronic viral hepatitis, which can cause cirrhosis, fibrosis, and hepatocellular carcinoma (HCC) [1][2][3]. Moreover, response to therapy is different and less satisfactory in patients with hepatitis delta virus (HDV) infection than hepatitis B virus (HBV) monoinfection [4]. It is estimated that 15 to 20 million HBV patients are positive for anti-HDV antibody [5]. The HDV incidence has declined in endemic countries in Western Europe such as Italy [6]. Hence, HDV persists as an applicable cause of morbidity in Eastern Europe and the Mediterranean basin [7][8]. In Iran, as a country located in the Mediterranean basin, the prevalence of HBsAg carrier is 2.14%, according to a recent review [9]. Different rates of HDV infection among HBsAg-positive patients have been reported from Iran. The first report on this issue was described by Malekzadeh et al. in asymptomatic hepatitis B carriers in Shiraz (South of Iran), in which HDV prevalence was 13.9% [10], but recent studies have shown that HDV rates varied from 0% in northern Iran [11] to 20% in southern Iran [12].

## 2. Objectives

To provide a clear estimate of HDV prevalence in Iran and address a gap in the field's knowledge, we designed a systematic review to collect all respective epidemiological studies conducted in Iran about HDV in chronic hepatitis B patients. The prevalence of HDV was analyzed separately in the setting of chronic hepatitis, liver cirrhosis, HCC, and inactive carriers to show the effects of HDV on hepatitis progression. We investigated regional differences as well as potential chronological changes to analyze epidemiological changes of hepatitis D in various parts of Iran. Pooled estimations for each possible risk factor, especially in high-risk groups, were calculated to identify the most important routes of HDV transmission.

## 3. Data Sources

The authors reviewed studies and evaluated the prevalence of HDV infection in HBsAg positive cases. The outcomes considered in this review were the prevalence and risk factors of HDV infection.

One author (N.A.) conducted an electronic literature search through Scopus, ISI, Google scholar, and three Medline database engines-PubMed, Embase, and Ovid-using different combinations of the word Iran and the key words "hepatitis D, Delta antigen, HDV, and hepatitis delta virus". Iranian databases, including MagIran, IranMedex, and SID, were also searched with relevant English and Persian key words. At the time when these searches were conducted, the databases were limited to published and unpublished information up to and including December 2010. Search sensitivity was checked by considering duplicated papers. If the full text of articles were not accessible, an e-mail was sent to the author. If no response was received after one month, the abstract was used to extract data (except for articles with no informative abstracts, which were omitted).

## 4. Study Selections

Only studies that clearly stated information about the numbers of HBsAg-positive patients infected with HDV were selected. Studies in which all patients had acute hepatitis B [13][14][15] were excluded because the pattern of HDV is different in chronic and acute hepatitis. Investigators also excluded articles that were about the genotypes of HDV. The names of the authors and journals did not impact exclusion.

### 4.1. Assessment of Study Quality

A critical appraisal (CA) was conducted using the Epible checklist [16] by three investigators (NA, SH SY, and AA SH) to evaluate the adequacy of sample size, research design, data collection, and presentation of results. If the investigators' scores were not close, they did conduct another CA together again. Based on the total CA score, articles were divided into low (< 40%), moderate (40 to 70%), and high (> 70%) quality. Low-quality papers were not included in the main analysis but were included in subgroup analyses.

## 5. Data Extraction

Data extraction was completed by three investigators (NA, SH SY, and AA SH) and rechecked by one of them (NA). Information was entered into Microsoft Office Excel 2007. The name of the city the author's name, year of study, HDV-detection method, sample size, HBsAg-positive frequency, mean age, and total prevalence of HDV were extracted. Moreover, standard errors (SE) were calculated as SE = √ (P × [1-P] / N), where P = prevalence and N = sample size. HDV prevalence was extracted in different subgroups consisting of cirrhotic patients, inactive carriers, chronic hepatitis patients, and male and female participants.

### 5.1. Statistical Analysis

A 95% CI of the seroprevalence of anti-HDV antibody was computed for each of the included studies using the approximate normal distribution model. The summary estimate of HDV prevalence was calculated as an average of the individual study results weighted by the inverse of their variances using fix/random models (DerSimonian and Laird) based on the heterogeneity test result using Q, I-squared and Tau-squared statistics. Due to the low power of this test, a minimum cut-off P value of 0.1 was established as a threshold of heterogeneity. I-squared lies between 0% and 100% and heterogeneity increases with increasing of I-squared value. Because few articles were available on some subgroups, Tau-squared is more suitable because it is not influenced by the number of studies. The results were expressed in geographic maps using Arc View 3.2 software (ESRI Inc., New York). For provinces with more than one study, the pooled estimation of anti-HDV prevalence was computed using the meta command, and then a survey-data analysis was used to estimate more accurate HDV-infection prevalence considering the weight of each city [17], which was calculated as the ratio of the city's HBV population to the study's sample size. The HBV population was calculated for each city by multiplying the city population [17] by the HBV-prevalence estimate [9]. The same method was used to calculate HDV prevalence for both genders.

Subgroup analyses were designed according to disease patterns (cirrhotic, chronic hepatitis, and inactive carriers) and quality assessment scores (good and moderate). An overall meta-analysis was performed for each risk factor to determine whether the factor increased HDV prevalence. The available data were used to calculate or confirm the unadjusted odds ratio (OR). Risk factors, without complete data to calculate their OR, were omitted. To make pooled estimates, the authors used the "metan" command to compute point-estimation ORs with a 95% CI for each risk factor. The analysis was performed with STATA 11 software (STATA Corp. LP).

## 6. Results

### 6.1. Studies

**Diagram 1:**
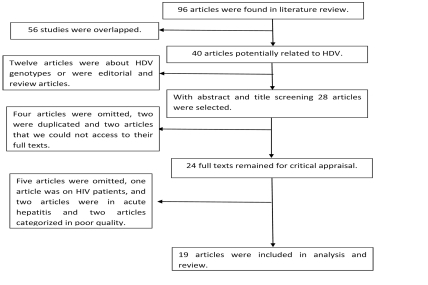
Article Selection Process for HDV Infection in Iran up to December 2010

Ninety-six articles were found in the literature review, 40 of which [4][10][11][12][13][15][18][19][20][21][22][23][24][25][26][27][28][29][30][31][32][33][34][35][36][37][38][39][40][41][42][43][44][45][46][47][48][49] were potentially related to HDV prevalence in Iran. The detailed search process is demonstrated in Diagram 1. Investigators sent an e-mail to the authors of four articles to obtain full text that was not available in the online databases [24][44][46][48]. Only two authors [24][44] responded within one month. After filtering the studies based on the inclusion criteria explained above, 19 studies were identified as assessing the prevalence of HDV infection in Iran. Out of these, four studies were conducted in Tehran [19][22][35][39], two studies in Shiraz and [10][45] Hamedan [21][23], and 1 each in the cities of Shahrekord [28], Mashhad [32], Khuzestan [33], Isfahan [24], Sari [11], Golestan [38], Babol [34], Bushehr [12], Kerman [50], and Tabriz [41]. Additionally, one study was carried out in both Tehran and Tabriz [44] Table 1. All of the studies in this report were based on cross-sectional study designs conducted between 1983 and 2009, and the sample sizes ranged from 16 to 1,725. The ages of the study subjects ranged from 24 to 43. All studies measured anti-HDV with individual patients' serums.

**Table 1:** Summary of Included Studies in Meta-Analysis of HDV Prevalence in Iran Between 1983 and 2000

**Geographic Area**	**First Author (y) (Citation) **	**Target Population**	**Age, mean**	**Sample Size**	**HDV Prevalence, %**	**Proportion a**	**Quality Assessment Score**
North							
Babol	Hassanjani Roshan et al. (2000-2) [34]	Asymptomatic HBsAg positive	6-75	546	2	184.0522	moderate
Golestan	Roshandel et al. (2004-5) [39]	Asymptomatic HBsAg positive	41	139	5.8	2489.616	good
Sari	Taghvaei et al. (2003-04) [11]	Asymptomatic HBsAg positive	35	167	0.00	634.7727	moderate
Tabriz	Seifi et al. (2006-07) [41]	Asymptomatic HBsAg positive	33	355	6.00	2048.32	moderate
Mashhad	Habibi et al. (2005-6) [32]	Not mentioned	39	200	9.00	2855.135	moderate
Center							
Isfahan	Ataei et al. (2009) [24]	Asymptomatic HBsAg positive	39	346	2.8	1228.67	good
Shahrekord	Doosti et al. (2003-4) [28]	Asymptomatic HBsAg positive	27	200	3	394.2586	moderate
Hamadan province	Amini et al. (1989) [23]	Asymptomatic HBsAg positive	24.4	123	2.40	2619.892	good
Hamedan City	Alizadeh et al. (2002-7) [21]	Asymptomatic and HBsAg positive	35.6	81	17.30	1683.741	moderate
Tehran	Rezvan et al. (1986-88) [39]	Asymptomatic and symptomatic HBsAg positive	43	238	2.50	10803.8	moderate
	Karimi et al. (2000) [35]	Asymptomatic HBsAg positive and Hemodialysis	-	219	8.70	7793.586	moderate
	Amini Kafi-abad et al. (2000) [22]	Asymptomatic HBsAg positive	42.2	79	8.80	21605	moderate
	Alavian et al. (2001-4) [19]	Asymptomatic and symptomaticHBsAg positive	39	280	5.70	6095.698	moderate
Tehran -Tabriz	Somi et al. (2007-8) [44]	Asymptomatic and symptomatic HBsAg positive	38.9	847	9.30	N.A^b^	moderate
South							
Kerman	Zahedi et al. (2006-7) [49]	symptomatic HBsAg positive	39.2	196	10.70	739.8832	good
Shiraz	Taghavi et al. (2003-4) [45]	symptomatic HBsAg positive	15-75	93	9.70	3937.568	good
Ahvaz	Malekzadeh et al. (1983) [10]	Asymptomatic HBsAg positive	33.3	158	13.90	1524.283	moderate
	Hajiani et al. (2002-8) [33]	Asymptomatic symptomatic and HBsAg positive	37	1725	11.50	166.0052	good
Bushehr	Makvandi et al. (2006) [12]	Cirrhotic patients	41	16	20	3013.347	moderate

^a^ proportion, City HBV population/sample size

^b^ N. A, Not Applicable

### 6.2. HDV Infection Prevalence

Reported HDV prevalence varied widely, from 0% in Sari [11] to 20% in Bushehr [12]. Based on the heterogeneity tests (Q = 20,967.40 df = 18, P <.001; I-squared = 99.9%), a random model was considered. Furthermore, Tau-squared indicated a variance of 18 between studies. The point estimation of HDV prevalence among 5,700 HBsAg-positive patients from 13 cities in Iran was 7.8% (95% CI: 5.89 - 9.71) from 1983 to 2009. According to the survey-data analysis, the HDV prevalence for each city was weighted using the HBV prevalence of the province's population divided by the sample size (Table 1). The weighted mean prevalence of HDV infection calculated from 4,853 participants was 6.61% (95% CI: 6.59 - 6.63). The total population of the cities considered in this survey was about 44% of the total population of Iran.

### 6.3. HDV Prevalence in Cirrhotic, Chronic-Hepatitis, Inactive-Carrier Patients

**Figure 1:**
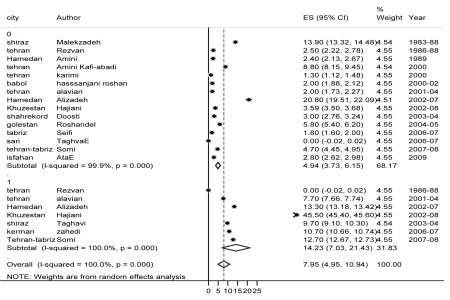
Forest plot of HDV Infection prevalence Among HBsAg-positive Patients in Iran, 1983-2008

One source of heterogeneity was the different target populations in the studies. Therefore, the point estimations were broken into three subgroups: cirrhotic, chronic-hepatitis, and inactive-carrier patients. There were 198 patients in the cirrhotic and HCC groups (4 articles: 4, 12, 33, 39), and the pooled estimation in this group was 30.47% (95% CI: 9.76 - 51.19). In chronic hepatitis group among 1,114 patients, the HDV prevalence in the random model was 14.4% (95% CI: 7.72 - 21.07; 7 articles: 19, 21, 33, 39, 45, 46, 50).In 4,372 inactive carrier patients, the HDV prevalence was 4.94 % (95% CI: 3.73 to 6.15; 15 articles: [10][11][19][21][22][23][24][28][33][34][35][38][39][41][44] Figure 1.

### 6.4. HDV Prevalence According to Different Article Quality

The subgroup analyses depended on the quality of the studies. The HDV prevalence in articles [21][23][24][33][45][48], which had good quality scores was 7.15% (95% CI: 3.14 to 11.14). In papers with moderate quality scores, the HDV prevalence was 8.09% (95% CI: 6.02 to 10.15). In low-quality studies, the HDV prevalence was 5.04% (95% CI: -4.65 to 14.75).

### 6.5. Gender Subgroup Analysis

The pooled estimations for women and men were calculated separately using a survey-data-analysis method. The prevalence of HDV was estimated at 8.63% (95% CI: 8.53 - 8.73) among 2,644 men and 5.34% (95% CI: 5.26 - 5.44) among 1,390 women.

### 6.6. Risk Factors

Ten articles [19][21][22][24][34][35][38][39][41][45] mentioned risk factors. Point estimations were calculated for factors such as history of blood transfusion, intravenous drug abuse, and hemodialysis and HBeAg positive serology. Table 2 demonstrates the relationship between different risk factors and anti-HDV serology.

**Table 2:** pooled HDV Risk Factors Among HBsAg positive patients in Iran, 1983-2008

	**Study (Investigation Year)**	**OR**	**95% CI**	**Weighting Score**	**Pooled Estimation (95% CI)**
Blood transfusion					1.1 (0.40- 2.98)
	Alizadeh et al. (2002-2007) [21]	0.43	0.05-3.73	0.84	
	Taghavi et al. (2003-2004) [45]	0.62	0.07-5.4	0.83	
	Alavian et al. (2001-2004) [19]	2.4	0.64-9.1	2.18	
HBeAg positive					1.26 (0.66-2.4)
	Ataei et al. (2009) [24]	0.72	0.15-3.4	1.56	
	Alizadeh et al. (2002-2007) [21]	2.2	0.66-7.22	2.70	
	Hassanjani Roshan et al. (2000-2002) [34]	5.1	1.47-18.33	2.42	
	Rezvan et al. (1986-1988) [39]	0.83	0.025-0.27	2.67	
	Amini Kafi-abad et al. (2000) [22]	0			
IDU					1.6 (0.78- 3.214)
	Taghavi et al. (2003-2004) [45]	5.92	1.36-25.78	1.77	
	Jedari Seifi et al. (2006-2007) [41]	2.45	1- 6.03	4.75	
	Alizadeh et al. (2002-2007) [21]	2.06	0.35 - 11.92	1.25	
Hemodialysis					1.72 (0.79- 3.76)
	Karimi et al. (2000) [35]	24.79	5.56 -110.48	1.72	
	Jedari Seifi et al. (2006-07) [41]	1.7	0.68- 4.23	4.61	
	Taghavi et al. (2003-2004) [45]	0			

## 7. Conclusions

According to our results, the overall estimation of HDV seropositivity in Iran is about 6.61% in HBsAg positive patients. The estimates were also run for asymptomatic and symptomatic HBsAg-positive patients. In our findings, the overall estimation in the asymptomatic group was about 5%, and the time trend did not follow a recognizable pattern. Without considering outliers, the HDV prevalence moved closer to 5%. The HDV prevalence among symptomatic HBsAg-positive patients was about 14.5%, and it has been increasing over the years. The increase in HDV prevalence among symptomatic HBsAg-positive is different from the reports of declining cases in Italy [50], Spain [51], Taiwan [52], and Turkey [53]. The declining incidence rates in these countries may be related to better detection. In addition, the HDV-infection rate in the Iranian population is lower in comparison with other countries in the Eastern Mediterranean Region [7][53], and it is nearer to the rates in European and East Asian countries (Table 3).

**Table 3:** prevalence of HDV in Different Regions and Comparison With HDV prevalence of Iran provinces

	**Country/Region**	**Target Population**	**Prevalence, %**	**Sample Size**	**Neighbors City in Iran**	**Prevalence in Neighbors, %**
Degertekin H et al. (2008) [53]	Turkey/ middle east	Meta-analysis	27.1	6734	Tabriz	6
Jacobson IM et al. (1985) [56]	Afghanistan/EMRO	high risk group and patients	28.6	362	Mashhad	9
Baig S et al. (2009) [57]	Pakistan/ EMRO	patients	37	129	Kerman	10.7
Al Tarif I et al. (2004) [58]	Saudi Arabia/EMRO	patients	8.6	19250	Khuzestan	11.5
Zaki S et al. (2010) [59]	Egypt/ EMRO	high risk group and patients	20	100	none	-
Gaeta GB et al. (2003) [50]	Italy/ Europe	14 referral liver unites	8.3	834	none	-
Chen X et al. (1998) [60]	China/ Asia	sample infected with HBV	7.72	2681	none

**Figure 2:**
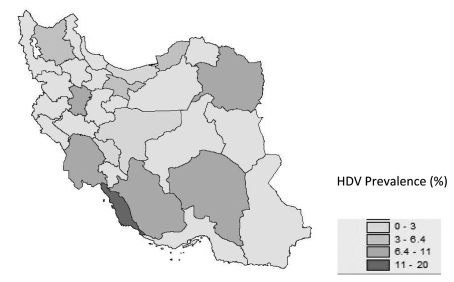
Regional Distribution of Pooled or Individual Prevalence of Hepatitis D Virus Infection Among HBsAg-positive patients in Iran, 1983-2008

HDV is more common in the south of Iran than in the north. However, HBV prevalence is higher in the north [9]. The difference in HDV rates may be due to factors that have an impact on HDV acquisition such as the generally lower socioeconomic status in south of Iran. Moreover, we have scarce data about HBV and HDV prevalence rates in southern Iran (Figure 2).

Our findings show that HDV is more common among cirrhotic and HCC patients. In a retrospective study in European patients with HBV-related cirrhosis, Fattovich et al. [55] found that the risk of HCC increased 3 times in HDV patients. In another study, during 233 months of follow-up [3], 82% and 15% of chronic HDV patients developed cirrhosis and HCC. This finding indicates that HDV causes a severe form of chronic hepatitis in comparison with HBV monoinfection. The longer history and more severe condition cause a higher rate of anti-HDV antibody, which is in line with previous studies.

In our study, the prevalence of HDV was higher in males (8.63%) than in females (5.34%). Blood transfusion was generally more common in women, but we could not detect this factor's impact on HDV prevalence. However, other factors, such as a greater possibility of multiple partners, intravenous drug abuse, war injury, and a higher rate of HBV infection in men [9], can be explained as possible causes of this divergence. The results of this study showed that the main routes of transmission for HDV are blood and blood products; therefore, individuals with a history of transfusion, surgery, tattooing, war injury, dentistry interventions, endoscopy, hemodialysis, intravenous drug use, and patients with coagulation factor disorder are at risk of HDV. This route of transmission is more similar to Western Europe and United states [56]. The important groups in our review were hemodialysis patients and intravenous drug users. However, this association was not statistically significant, which may be due to the unadjusted ORs to others factors. We did not have enough data to assess interfamilial and sexually transmitted routes. In addition, the trend of the disease in HBeAg-positive patients with hepatitis D has not been well established. Previous articles showed HBeAg-positive rates of 15 to 30% among HDV patients [6]. Our findings demonstrate that HDV is more common in patients who are HBeAg positive.

This review has some limitations, such as the lack of library and thesis searches. Additionally, data were available from 43% of provinces, and most of the data came from urban areas. The common method used for HDV detection was ELISA. Confirmation of ongoing HDV infection by PCR testing of HDV RNA was missing. The impact of this lack of information was that patients with and without active delta infection could be differentiated. The strongest part of this study was the use of a survey-data analysis in addition to the usual meta commands to generalize the results to the whole population. Moreover, a critical appraisal allows for more accurate estimates. A quality subgroup analysis showed that the low-quality papers underestimated HDV prevalence.

In conclusion, the prevalence of HDV is less common in Iran than in endemic regions; however, this is a severe form of hepatitis in Iranian patients. The most probable route of HDV transmission is hematologic, which shows the importance of blood screening for HDV, especially in groups with numerous blood transfusions. Information is lacking from some provinces, and ongoing research is required to understand the effects of HDV infection on HBsAg-positive patients and its risk factors. More research should be conducted on HDV to develop innovate strategies to control and diagnose this most severe form of viral hepatitis.
